# Comparison of CURB-65 and CRB-65 as predictors of death in community-acquired pneumonia in adults admitted to an ICU

**DOI:** 10.1186/cc12655

**Published:** 2013-06-19

**Authors:** AR Santana, FF Amorim, FB Soares, LG de Souza Godoy, L de Jesus Almeida, TA Rodrigues, GM de Andrade Filho, TA Silva, OG da Silva Neto, PHG Rocha, PN Ferreira, APP Amorim, E Bastos de Moura, JA de Araújo Neto, M de Oliveira Maia

**Affiliations:** 1 Unidade de Terapia Intensiva Adulto do Hospital Santa Luzia, Asa Sul, Brasília, DF, Brazil

## Introduction

Community-acquired pneumonia is one of the most common causes of sepsis and ICU admissions. Patients with CAP who demand critical care had mortality rates of 25 to 50%. Thereby, the assessment of the severity is essential to guide the treatment. There are several severity scores for CAP and some of the most acknowledged are CURB-65 and CRB-65. The objective of this study was to evaluate the accuracy of CURB-65 and CRB-65 as predictors of death in patients with community-acquired pneumonia.

## Methods

A prospective study during 6 months was conducted with patients diagnosed with CAP admitted to the ICU of the Hospital Santa Luzia, Brasília, DF, Brazil. Patients were stratified according to CURB-65 (0 to 5) and CRB-65 (0 to 4) and their risk categorized as: low (CURB-65: 0 to 1 and CRB-65: 0), moderate (CURB-65: 2 and CRB-65: 1 to 2) and high (CURB-65: 3 to 5 and CRB-65: 3 to 4). The sensitivity, specificity, positive predictive value (PPV), negative predictive value (NPV), likelihood ratio positive (LR+), and likelihood ratio negative (LR-) were calculated. Validity and reliability were assessed with the Spearman correlation coefficient. Patients with chronic kidney failure and those submitted to mechanical ventilation at the time of admission were excluded.

## Results

**Figure 1:**
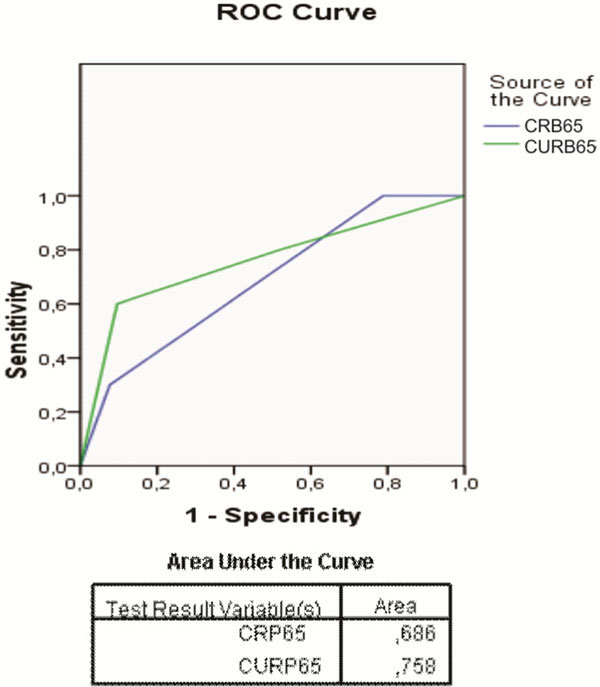


A total of 62 patients were included. Twenty-seven with low risk, 24 with moderate risk and 11 with high risk according to CURB65 and their mortality rates were 7.4%, 8.3% and 54.5%, respectively. According to CRB-65, 11 were low risk, 44 moderate risk and seven had high risk. The mortality on CRB-65 stratification was 0%, 15.9% and 42.9% for low, moderate and high risks, respectively. When we gathered moderate and high risks, CRB-65 was more sensitive (1.00 vs. 0.80) and had better LR- (0.00 vs. 0.41), and NPV (1.00 vs. 0.92). CURB-65 had better specificity (0.48 vs. 0.21), LR+ (1.54 vs. 1.26), and PPV (0.23 vs. 0.20). The receiver operating characteristic curves of CURB-65 and CRB65 had areas of 0.758 and 0.686, respectively. The Spearman correlation coefficient was 0.612 (*P *= 0.00). See Figure 1.

## Conclusion

CURB-65 and CRB-65 had a high correlation. CRB-65 was more sensitive as a predictor of death as well as a guidance for hospitalization. Moreover, CRB-65 is a more practical score since it does not use laboratorial variables.
